# Medical resident training in China

**DOI:** 10.5116/ijme.5ad1.d8be

**Published:** 2018-04-26

**Authors:** Sheng-Li Huang, Qi Chen, Ying Liu

**Affiliations:** 1 Second Affiliated Hospital, College of Medicine, Xi'an Jiaotong University, Xi'an, China; 2 School of Foreign Studies, Xi'an Jiaotong University, Xi'an, China

**Keywords:** Medical, resident, training, medical education, China

## Introduction

Medical education is key for the successful development of a health system in a country.^1^ In China, medical education starts after high school and varies from three to six years at the undergraduate level, followed by additional years at the graduate level. In most medical universities in China, the undergraduate curriculum comprises four years of basic courses and clinical sciences, followed by one year of a clinical internship. The internship involves rotations in various departments; each rotation lasts for a couple of months. After graduation, most of the graduates who get jobs in hospitals or clinics will work as resident physicians for a specified period. During this period, they learn from experienced physicians, further develop their knowledge and skills, and get familiar with the working environment. This period is termed resident training.

Medical resident training is internationally recognized as a continuing medical education process, aiming to cultivate the ability of novice physicians to work independently. Training of the residents is thus crucial to the quality and development of medical services.^2^^-^^4^ In China, the resident training program has experienced necessary changes in the past few decades to cope with the drastic increase in people's needs of high-quality medical services as well as the rapid development of medical science and technology. Given the fact that the world today is increasingly globalized and local health care resources have to address the needs of not only residents but also immigrants and visitors from other parts of the world, it is valuable to introduce medical resident training in China to an international audience. In this way, the rest of the world may develop some understanding of our situation, and at the same time, we can learn from their feedback to further improve our programs. To our knowledge, there are few articles regarding Chinese medical resident training. This article provides an overview of the development of the training programs in China with a purpose to raise attention and elicit opinions from international medical and educational professionals.

### Resident training in the past

The emergence of medical resident training in China dates back to the early 1920s. Peking Union Medical College Hospital, one of the early medical colleges in China, set up the first residency program in China in 1921.^5^ However, the development of a residency program did not go off smoothly. Before the establishment of the People's Republic of China in 1949, medical education in the country remarkably lacked due to the chaos and poverty caused by wars.^6^ After 1949, the medical education system was gradually taking shape. However, the Cultural Revolution from 1966 to 1976, a decade of internal turmoil, disrupted the normal function of medical institutions and resulted in essential and nationwide cease of medical education.^7^

A new historical era was ushered in after the end of the Cultural Revolution. In 1979, the Ministry of Health (MOH) issued the Trial Assessment of Resident Training in Hospitals Affiliated to Medical Colleges. Since then, resident training has been officially acknowledged and institutionalized. Some areas were selected for the trial of the training system. The affiliated hospitals of some medical colleges in these areas set up training programs which aimed to equip medical graduates with specialized knowledge in the field of clinical medicine. The trials were performed only in hospitals affiliated to medical colleges but not in other types of hospitals. Also, the resident training for becoming a clinical doctor was abbreviated and neglected.

In 1993, MOH reorganized and issued the Trial Measures for Standardized Resident Training of Clinical Residents. Since then, all second-level grade-A hospitals and third-level hospitals set up resident training programs under the MOH guidelines. The primary objective of the programs, in this period, was to provide continuing education that turned medical graduates into qualified clinical physicians. One prominent characteristic of this period is that resident training in different hospitals varied considerably. Not every hospital provided a training program.

Hospitals that ranked higher usually offered more and better training opportunities. There was not yet a unified standard for resident training and evaluation at the national level. In this situation, the continuing education of medical graduates relied largely on what hospitals they worked with. If a graduate was employed by a high-level hospital, he would be likely to receive comprehensive and organized training at the position of a resident physician, which would help significantly in his development of professional knowledge and skills. In contrast, those in hospitals where a well-organized residency program was not available would have no such chances and have to rely on the experience learned at college or through an internship. This means that graduates who had gone through the same or similar curriculum at college might develop very differently in their post-graduation professions due to the differences between the hospitals where they worked. This difference might further influence the balance in the distribution of medical resources and the quality of health service as a whole.

### Resident training in the present

With the rapid development of health service and medical education in recent years, the need for national standards to ensure the quality of residency programs has been recognized. In December 2013, seven Chinese government ministries jointly launched the Guidelines for Standardized Resident Training (SRT). The release and implementation of the guidelines represent China's commitment to achieving its quality standards as a national strategy. The guidelines will become compulsory nationally by 2020.

The guidelines for SRT include four sections: training objective, rotation length requirement, training content, and reference material.^8^

The aim of resident training, according to SRT, is to equip medical graduates with practical clinical skills and enable them to become application-oriented, multi-skilled professionals serving in the national health system. Different from previous guidelines, SRT is focused mainly on cultivating qualified practitioners rather than academics.

Medical graduates, after successful completion of schooling, are required to take SRT in their chosen specialties. After taking SRT, they are enrolled in the nationally recognized continuing education program. Based on SRT, graduates need a minimum of three years working as a resident physician.^9^

The guidelines list in great detail the diseases to be familiar with and the skills to master in each of the medical specialties. Residents are actively involved in patient assessment and all aspects of patient care, such as initial history and physical, diagnosis, therapeutic planning, and interaction with patients and their families. They play essential roles in the daily activities in clinics and hospitals.

The year 2014 marks the beginning of standardized medical resident training in China. Since the launch of SRT in the preceding year, the guidelines have been implemented, boosting rapid development of resident training nationwide.

Currently, taking SRT is obligatory in the national health care system, which ensures that all hospitals in all regions carry out residency programs following the same standards. This practice guarantees the consistency and further the quality of resident training across the country. The training is typically provided by government-accredited training bases, such as tertiary grade-A hospitals, due to their affordances in terms of human resources, equipment, financial support, and disease cases. The Chinese Medical Doctor Association has been designated to manage the accreditation of training bases. A subsidiary branch of the association is in operation in altogether 31 provinces, municipalities, and autonomous regions in China. The unified accreditation of training bases has guaranteed the consistency and quality of the resident training programs nationwide.

Trainees are evaluated through a series of quantitative and qualitative assessments provided by MOH. Trainees who complete the training process and pass a demanding examination are awarded a certificate.

It is true that the actual operation of residency training differs slightly across hospitals due to the different characteristics and conditions of these medical service organizations. However, the standardized training system guarantees, to its utmost, the consistency in the content and quality of resident training nationwide. Most trainees agree that the program has enhanced their clinical capacity and paved the way to their professional development.

### Resident training in future

A residency program is a key to subspecialty training. It is the essential and almost only means through which medical graduates get trained in the subspecialty of their career choice. It is the step that students have to take to transform from one with medical knowledge in general to a professional with knowledge, skills and experience specific to the subfield that he picks to serve in. This training process is highly associated with the quality of medical human resources in the country. A standardized training program is thus important to the overall healthcare and medical services.

In January 2016, MOH issued a regulation entitled Instruction for Trial Measures for Standardized Specialist Training, which specifies guidelines and policies for training specialized physicians.

Currently, subspecialty training is being piloted in some developed regions such as Shanghai municipality, but resistance and challenges may exist in the implementation of the training. Trainees have diverse needs and interests, which might not be thoroughly or specifically addressed. Issues exist in the training processes, such as the concern of patient safety in surgical procedures where trainees need to be involved. Further studies are required to explore how to perform the training without putting patients at risk or reducing treatment quality. The quality and consistency of supervision is another issue that needs to address. Ideally, each trainee should be supervised closely by responsible senior doctors. But currently, there is a lack of evaluation and control of the training supervision. It is not clear if the senior doctors have performed their responsibilities successfully as required. An effective, thorough system that manages, supervises and regulates the training processes is urgently needed.

## Conclusions

Remarkable development has been made in the resident training in China, but considerable challenges exist and much remains to be done. The country is making significant effort to improve its medical system. The drastic economic growth in the country facilitates the development of the system, and the improved living standard of people also calls for better health care services. To sum up, China is striving to improve its health care system. China is the world's largest developing country with a large body of medical graduates who are facing SRT. The problems regarding SRT in China may also exist in other developing countries. The movements and changes in this country, therefore, may be referential to other developing countries. What's more, China hosts numerous foreign visitors, job seekers, and immigrants, and the numbers of these people are increasing every year. There is in need to develop the medical system to serve the international population in this country. It is time for the country to open up and introduce in advanced theories and practices in the medical resident training system.

### Acknowledgements

This work was supported by a research grant from the Pilot Project of Comprehensive Reform of Postgraduate Education in Shaanxi Province, China.

### Conflict of Interest

The authors declare that they have no conflict of interest.
